# Supplementary material for: Comparative multiomics analysis of cell physiological state after culture in a basket bioreactor
Source: Sci Rep. 2022 Nov 23;12:20161. doi: 10.1038/s41598-022-24687-4 (PMC9686226; doi:10.1038/s41598-022-24687-4)
Supplement: Supplementary file 1 — Supplementary Information 1. [file 41598_2022_24687_MOESM1_ESM.zip › raw data/Transcriptomic raw data/6.Enrichment/2.KEGG/TE1vsTC1/GSEA/index.html]

Index for xtools.gsea.Gsea TE1vsTC1.Gsea.1656483128235

### GSEA Report for Dataset readcount

#### Enrichment in phenotype: **TE1 (2 samples)**

- 39 / 84 gene sets are upregulated in phenotype **TE1**- 0 gene sets are significant at FDR < 25%- 12 gene sets are significantly enriched at nominal pvalue < 1%- 12 gene sets are significantly enriched at nominal pvalue < 5%- Snapshot of enrichment results- Detailed enrichment results in html format- Detailed enrichment results in excel format (tab delimited text)- Guide to interpret results

#### Enrichment in phenotype: **TC1 (2 samples)**

- 45 / 84 gene sets are upregulated in phenotype **TC1**- 0 gene sets are significantly enriched at FDR < 25%- 12 gene sets are significantly enriched at nominal pvalue < 1%- 12 gene sets are significantly enriched at nominal pvalue < 5%- Snapshot of enrichment results- Detailed enrichment results in html format- Detailed enrichment results in excel format (tab delimited text)- Guide to interpret results

#### Dataset details

- The dataset has 22403 features (genes)- No probe set => gene symbol collapsing was requested, so all 22403 features were used

#### Gene set details

- Gene set size filters (min=0, max=5000) resulted in filtering out 3 / 87 gene sets- The remaining 84 gene sets were used in the analysis- List of gene sets used and their sizes (restricted to features in the specified dataset)

#### Gene markers for the **TE1** *versus* **TC1** comparison

- The dataset has 22403 features (genes)- # of markers for phenotype **TE1**: 21031 (93.9% ) with correlation area NaN%- # of markers for phenotype **TC1**: 1372 (6.1% ) with correlation area 0.0%- Detailed rank ordered gene list for all features in the dataset- Heat map and gene list correlation  profile for all features in the dataset- Buttefly plot of significant genes

#### Global statistics and plots

- Plot of p-values *vs.* NES- Global ES histogram

#### Other

- Parameters used for this analysis

#### Comments

- Timestamp used as random seed: 1656483128410- Warning: Phenotype permutation was performed but the number of samples in class A is < 7, phenotype: sample.cls- Warning: Phenotype permutation was performed but the number of samples in class B is < 7, phenotype: sample.cls- With small datasets, there might not be enough random permutations of sample labels to generate a sufficient null distribution. In such cases, gene\_set randomization might be a better choice.

---

Report: TE1vsTC1.Gsea.1656483128235.rpt   by user: liuyan6860

xtools.gsea.Gsea [Wed, Jun 29, '22 2 PM 12]

Website: www.gsea-msigdb.org/gsea
Questions & Suggestions: Contact page
